# ABO Blood Group. Related Investigations and Their Association with Defined Pathologies

**DOI:** 10.1100/tsw.2007.133

**Published:** 2007-08-10

**Authors:** Ursula Jesch, P. Christian Endler, Beatrix Wulkersdorfer, Heinz Spranger

**Affiliations:** Interuniversity College for Health and Development Graz, Castle of Seggau, Austria

**Keywords:** blood group type, ABO blood system, pathologies, connection, Germany

## Abstract

The ABO blood group system was discovered by Karl Landsteiner in 1901. Since then, scientists have speculated on an association between different pathologies and the ABO blood group system. The aim of this pilot study was to determine the significance between different blood types of the ABO blood group system and certain pathologies. We included 237 patients with known diagnosis, blood group, sex, and age in the study. As a statistical method, the Chi-square test was chosen. In some cases, a significant association between the blood groups and defined diseases could be determined. Carriers of blood group O suffered from ulcus ventriculi and gastritis (X1 = 78.629, p <0.001), colitis ulcerosa and duodenitis (X1 = 5.846, p < 0.016), whereas male patients carrying blood group A tended to contract different types of tumours. In patients with intestinal tumours, females with blood group A were more likely to develop the pathology, whereas in males, the blood group O dominated. The development of cholelithiasis was found, above all, in patients with blood group O, which differed from other research where a correlation between this pathology and blood group A was found.

## INTRODUCTION

The ABO blood group system was found by Karl Landsteiner at the University of Vienna in 1901, while investigating the reason why blood transfusions caused death in some cases, but saved lives in others[1,2]. Since then, scientists, such as Helmbold[3,4,5,6,7], Böckmann[8], Küpferling[9], Vogel and Krüger[10,11], Bernd and Pietschker[12], have searched for an association between different pathologies and the ABO blood group system of patients. The ABO system occurs as a result of polymorphism of complex carbohydrate structures of glycoproteins and glycolipids expressed at the surface of erythrocytes or other cells, or present in secretions, as glycan units of mucin glycoproteins. The blood types are inherited through genes on chromosome 9[13].

The ABO gene is autosomal and, therefore, every person carries two copies of genes coding for their ABO blood group. The A and B blood groups were dominant over the O blood group and their genes were codominant[2,13]. In fact, if a person inherited one A group gene and one B group gene, his/her red cells would possess both A and B antigens. The alleles were termed A (production of A antigen), B (production of B antigen), and O (no antigen production)[14].

The aim of this study was to determine if there is any significant association between blood groups and certain kinds of diseases, and, furthermore, if the results of this study correlate with the findings of other research groups.

## METHODS

All the data for this study were derived from the Surgical Clinic of the University of Leipzig and contain case histories from patients within a time frame of 1 year (2002). Only patients with known clinical diagnoses, blood group, age, and sex were included; 237 patients participated in the study. Table 1 shows a summary of the different groups of pathologies and the numbers of patients.

**TABLE 1**

| Disease | Patients (N) |
|---|---|
| Ulcus ventriculi and gastritis | 22 |
| Colitis ulcerosa and duodenitis | 17 |
| Gastric cancer | 24 |
| Struma nodosa | 33 |
| Cholelithiasis | 49 |
| Rectal cancer | 19 |
| All intestinal tumours (including rectal cancer) | 44 |
| Tumours (without breast cancer) | 93 |
| Breast cancer | 23 |

Table 2 describes the distribution of the blood groups of the patients included in the study.

**TABLE 2**

| Blood Group | Distribution in % |
|---|---|
| BG A | 38.4 |
| BG 0 | 40.9 |
| BG B | 15.6 |
| BG AB | 5.1 |

### Statistical Analysis

A statistical analysis was performed regarding the relation between several diagnoses and the blood group A, O, B, and AB. Males and females were treated as two separate, as well as one, group (see Table 3).

Based on the investigations by Ludwig[15], Lorenz[16], and Helmbold[5,6], who found significant associations between defined pathologies and blood type A and O, we decided to perform an additional analysis considering only blood group A and O. Also in this case, males and females were treated as two separate, as well as one, group.

**TABLE 3**
**Men and Women Together Considering all Blood Types**

| Disease | Expected Probability (%) | Real Probability (%) | | | | $\chi^2_3$ | *p* |
|---|---|---|---|---|---|---|---|
| | | BG A | BG O | BG B | BG AB | | |
| Ulcus ventriculi and gastritis | 7.3 | 2.9 | 13.9 | 8.9 | 0.0 | 62.466 | < 0.001 |
| Colitis ulcerosa and duodenitis | 5.7 | 3.6 | 5.9 | 8.9 | 12.5 | 62.466 | < 0.001 |
| Gastric cancer | 8.0 | 7.2 | 4.0 | 17.8 | 12.5 | 120.328 | < 0.001 |
| Struma nodosa | 11.0 | 11.6 | 13.9 | 2.2 | 12.5 | 93.861 | < 0.001 |
| Cholelithiasis | 16.3 | 10.1 | 25.7 | 17.8 | 6.3 | 174.379 | < 0.001 |
| Rectal cancer | 6.3 | 8.7 | 3.0 | 8.9 | 0.0 | 118.347 | < 0.001 |
| All intestinal tumours (including rectal cancer) | 14.7 | 15.9 | 10.9 | 24.4 | 0.0 | 279.189 | < 0.001 |
| Tumours (without breast cancer) | 24 | 23.9 | 26.7 | 20.0 | 18.8 | 22.731 | < 0.001 |
| Breast cancer | 18.0 | 12.7 | 14.3 | 31.8 | 33.3 | 206.115 | < 0.001 |

All patients with the same defined pathology according to Table 1 were considered as one group. Within the groups, the probability of developing a special pathology in patients with a special blood group was determined. The Chi-square test was used. Its value for the different diagnoses in relation to blood groups provided information about a significance in the association between the incidence of a special disease and a special blood group (see Table 3).

## RESULTS

A significant association between the blood groups and defined pathologies was found in some cases, which corresponded to the findings of others[11,12,16]. It was more likely that a carrier of blood group O suffered from ulcus ventriculi and gastritis ($\chi^2 1 = 78.629$, $p < 0.001$), colitis ulcerosa and duodnitis ($\chi^2 1 = 5.846$, $p < 0.016$), whereas male patients carrying blood group A tended to contract different types of tumours.

In the group of patients with intestinal tumours, the results differed in males and females. Women with blood group A were more likely to develop an intestinal tumour, whereas in man, the blood group O dominated. Additionally, development of cholelithiasis was found, above all, in patients with blood group O. In this case, our findings did not match those from other authors, such as Böckmann[8] and Küpferling[9], who described a correlation between this pathology and blood group A.

## DISCUSSION

The aim of this study was to determine a relationship between the blood groups and surgically treated pathologies. Our data suggest that there seem to be pathologies that occur with a certain preference in people with a special blood group. The number of patients (n = 237) included in our pilot study was fairly small. Since all our data were exclusively taken from the surgical care unit, the mentioned diagnoses were limited, but underwent histological examinations.

Other authors have also hypothesised that the presence of a certain kind of pathology may correlate with a special blood group. For example, it has been reported that blood group A seems to have an association with oral pathologies, such as dermatophytosis[18]. All these studies included only two aspects, the pathology and the blood type. No further information about the individual patient was considered.

According to our findings, further differentiations should be considered and a prospective study seems to be a meaningful instrument. With regard to this, we accept the statement of Bakare et al.[19] that varieties of ABO may play an important role in immunology and in prevention of diseases.
